# Diel, seasonal and vertical changes in the abundance, biomass and community structure of pelagic polychaetes at the subtropical station S1 in the western North Pacific: comparison with the results from the subarctic station K2

**DOI:** 10.1093/plankt/fbad023

**Published:** 2023-06-13

**Authors:** Kanako Amei, Ryo Dobashi, Minoru Kitamura, Atsushi Yamaguchi

**Affiliations:** Faculty/Graduate School of Fisheries Sciences, Hokkaido University, 3-1-1 Minato-cho, Hakodate, Hokkaido 041−8611, Japan; Department of Oceanography, University of Hawai‘i at Mānoa, 1000 Pope Road, Honolulu, HI, USA; Research Institute for Global Change, Japan Agency for Marine-Earth Science and Technology, 2−15 Natsushima-cho, Yokosuka, Kanagawa 237−0061, Japan; Faculty/Graduate School of Fisheries Sciences, Hokkaido University, 3-1-1 Minato-cho, Hakodate, Hokkaido 041−8611, Japan; Arctic Research Center, Hokkaido University, Kita-21 Nishi-11 Kita-ku, Sapporo, Hokkaido 001−0021, Japan

**Keywords:** annelida, K2S1 project, species diversity, vertical distribution, water mass

## Abstract

Information on pelagic polychaete community structure in the western North Pacific is available for the subarctic region (Station K2) but not for the subtropical region. Hence, we analyzed day–night vertically stratified samples collected in eight layers within the first 1000 m of the water column during four seasons in 1 year, using the same sampling method as St. K2, at the subtropical region (Station S1). At St. S1, 27 species of pelagic polychaetes belonging to 13 genera and six families were identified. The annual mean abundance was 35.0 ind. 1000 m^−3^ and the biomass was 17.3 mg WW 1000 m^−3^. At St. S1, the numbers of genera and species were higher and the annual mean abundance and biomasses were much lower than St. K2. The pelagic polychaetes often peaked in the mesopelagic layer at St. K2, with the carnivores and particle feeders peaking in the epipelagic and mesopelagic layers, respectively. At St.S1, the carnivorous species predominated throughout the entire water column, and were most abundant in the epipelagic layer. Thus, In the western Pacific Ocean, the subarctic pelagic polychaete community structure changed vertically with feeding ecology. On the other hand, the subtropical community may be adapted to conditions of high irradiance and light transmission.

## INTRODUCTION

Pelagic polychaetes are widely distributed from the surface to the deep layers of oceans worldwide (Halanych *et al.*, 2007; Ushakov, 1974). Pelagic polychaete diets differs widely among species: herbivorous (Day, 1967; Hopkins and Torres, 1989), carnivorous (Feigenbaum, 1979; Lebour, 1922; Rakusa-Suszczewski, 1968) and detritivorous (Christiansen *et al.*, 2018; Robison *et al.*, 2010; Uttal and Buck, 1996). Thus, understanding accurately the role of pelagic polychaete in food webs and vertical material flux of marine ecosystems worldwide requires information at the species level. However, our knowledge of the ecology of pelagic polychaetes, especially their community structure, is scarce compared to their importance.

The western North Pacific has two large current systems: cold Oyashio (subarctic) and warm Kuroshio (subtropical). The subarctic and subtropical regions of the western North Pacific have distinct physical, chemical and biological characteristics. Two time-series observation stations, subarctic St. K2 and subtropical St. S1, were set by the Japan Agency for Marine-Earth Science and Technology (JAMSTEC) as representative stations for each area (Honda *et al.*, 2017). Various projects have been conducted in subarctic and subtropical regions, such as VERTIGO (Buesseler *et al.*, 2007; Steinberg *et al.*, 2008) and K2S1 (Honda *et al.*, 2016, 2017). Through these projects, various biological aspects such as primary production (Elskens *et al.*, 2008; Matsumoto *et al.*, 2014, 2016), phytoplankton standing stock (Boyd *et al.*, 2008; Fujiki *et al.*, 2014), bacterial communities (Kaneko *et al.*, 2016) and zooplankton communities (Kitamura *et al.*, 2016; Kobari *et al.*, 2016; Steinberg *et al.*, 2008) have been evaluated.

Regarding pelagic polychaetes in the Pacific Ocean, regionally different distributions of genera and species have been reported, especially between the subarctic and subtropical regions (Tebble, 1962). The subarctic–subtropical comparisons have been carried out by analyzing zooplankton swimmer samples, known to swim actively (not just sink), collected by sediment traps moored at a depth of 200 m (Amei *et al.*, 2020), reporting seasonal abundances (fluxes) and population structures of the dominant species. Time-series surveys are essential to reveal the effect of environmental factors on the community structure of pelagic polychaete; however, no study has compared the time-series changes in the vertical structure between subarctic and subtropical regions. Regarding the community structure of pelagic polychaetes at St. K2 located in the western subarctic Pacific, diel, vertical and seasonal changes in community structure have been reported based on the analysis of day–night vertical stratified samples collected from the 0–1000 m water column in four seasons covering 1 year (Amei *et al.*, 2021). These findings are important for comprehensively evaluating diel, vertical and seasonal changes in pelagic polychaete communities from the surface to the deep sea. However, there are few comparable studies from other regions. At St. S1, JAMSTEC’s subtropical time-series station, zooplankton samples were collected using the same methods as at St. K2 (Kitamura *et al.*, 2016). However, the polychaete abundance, biomass and community structure at St. S1 have not yet been analyzed.

Hence, in this study, we analyzed pelagic polychaetes in the samples collected by day–night vertically stratified sampling to a depth of 1000 m during four seasons in 1 year at St. S1 in the western Pacific Ocean. We compared the results of this study with those reported in the study by Amei *et al.* at St. K2 in the western Pacific Ocean (Amei *et al.*, 2021), who applied the same methods as those applied in this study. This comparison may reveal the regional differences in polychaete communities between the subarctic and subtropical regions of the western North Pacific Ocean and the factors affecting them.

## METHODS

**Fig. 1:**
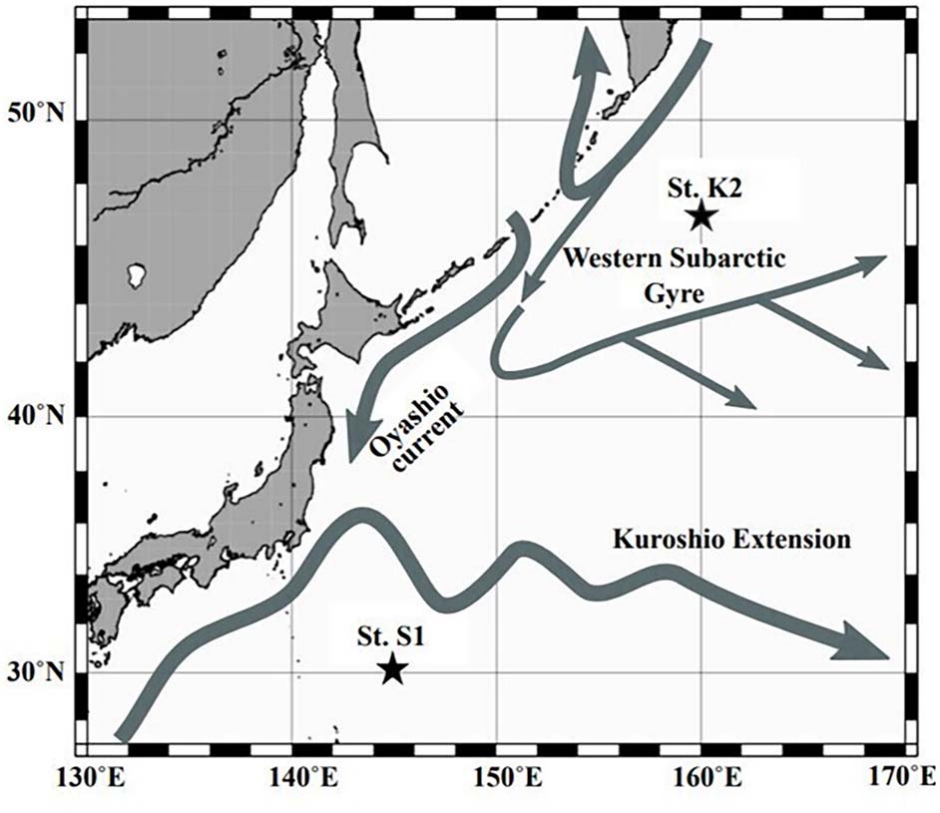
Location of the sampling stations: St. S1 (30°N, 145°E) in the subtropical western North Pacific Ocean. For comparison, the location of the subarctic station (St. K2) is also shown. Approximate positions of the currents are superimposed (cf. Yasuda, 2003).

Zooplankton samples were collected from eight layers of a 0–1000-m water column (0–50, 50–100, 100–150, 150–200, 200–300, 300–500, 500–750 and 750–1000 m) by oblique tows of the Intelligent Operative Net Sampling System (IONESS, SEA Corporation) (mesh size, 335 μm; mouth area, 1.5 m^2^) at St. S1 (30°00’N, 145°00′E; bottom depth, 5 800 m; Fig. 1) in the western Pacific Ocean during the days and nights of November 2010 and February, May and July 2011 (Table I). Temperature, salinity, dissolved oxygen (DO) and fluorescence of each sample were measured using a Conductivity, Temperature and Depth profiler (SBE 911 plus; Sea-Bird Electronics Inc.). The zooplankton samples were preserved in 4% (v/v) borax-buffered formaldehyde. See Kitamura *et al.* (2016) for detailed information on sampling.

**Table I:** Zooplankton sampling data (eight vertical stratification samplings between 0 and 1000 m) at St. S1 in subtropical western North Pacific from November 2010 to July 2011. D: day, N: night

Sampling date	Local time	D/N
10 November 2010	22:01–0:04	N
12 November 2010	12:09–14:10	D
15 February 2011	22:15–23:51	N
17 February 2011	0:00–0:05	N
19 February 2011	11:52–13:58	D
1 May 2011	21:58–23:51	N
2 May 2011	11:59–13:53	D
29 July 2011	12:49–14:46	D
29 July 2011	21:43–23:47	N
		

Based on temperature and salinity, the water masses were identified at each depth and sampling date. Throughout the year, we classified five water masses at St. S1: seasonal pycnocline water (SPW), subtropical mode water (STMW), transition water (TW), upper North Pacific intermediate water (upper NPIW) and lower North Pacific intermediate water (lower NPIW), based on the T–S characteristics at each sampling layer (Masuzawa, 1969; Yasuda, 2003; Yasuda *et al.*, 2001). SPW is the water above the pycnocline and is thus susceptible to solar radiation. STMW corresponds to the water of the remnant winter mixed layer in the subtropical North Pacific and has a temperature of 16–18°C ⁓100–300 m depth (Masuzawa, 1969). The water between STMW and NPIW was defined as TW in this study. A salinity minimum near 26.8 σθ characterizes NPIW. This water originates from the Okhotsk Sea and the western subarctic gyre (WSG) and flows toward the Kuroshio extension region (Yasuda, 2003). The upper NPIW (density centered at 26.6 σθ–26.9 σθ) and lower NPIW (density centered at 27.0 σθ–27.4 σθ) were more strongly affected by the water in the Okhotsk Sea and WSG, respectively (Yasuda *et al.*, 2001).

We sorted pelagic polychaetes from 1/4th of the bottle samples collected at each depth layer in the land laboratory. The specimens were identified to species and counted under a stereomicroscope. Species identification was performed to the lowest level possible, following Dales (1957) and Tebble (1962). For each genus or species, a batch of specimens in each sample was placed on a pre-weighed mesh, and the water was removed with the aid of wiping paper. Then, the wet weight (WW) was measured using an electronic balance (Mettler Toledo AT261) with a precision of 0.01 mg. The vertical distributions of the total abundance (ind. 1000 m^−3^) and total WW (mg 1000 WW m^−3^) were then quantified. For each sample, the Shannon-Weaver diversity index (*H′*) was calculated according to abundance and biomass using the following equation:


(1) \begin{equation*} {\displaystyle \begin{array}{c}{\boldsymbol{H}}^{\prime }=-\sum_{\boldsymbol{i}=\mathbf{1}}^{\boldsymbol{S}}{\boldsymbol{p}}_{\boldsymbol{i}}\times \mathbf{\ln}{\boldsymbol{p}}_{\boldsymbol{i}}\end{array}} \end{equation*}

Where *S* is the number of species, and *p_i_* is the relative dominance of specie*s i* (Shannon and Weaver, 1949). To determine the community structure of pelagic polychaetes, we calculated the similarity of samples based on abundance using the Bray–Curtis method (Bray and Curtis, 1957). The abundance data of pelagic polychaete species (*X*: ind.1000 m^−3^) were log-transformed (log [*X* + 1]) before analysis. A cluster analysis connected to complete linkage methods was performed for sample grouping. Nonmetric multidimensional scaling (NMDS; Minchin, 1987) and Pearson’s multidimensional coefficient analysis analyzed the relationships between environmental variables (depth, temperature, salinity, DO and fluorescence) and polychaete sample ordinations. To evaluate the effects of the seasons, day–night cycles and depth on the similarities between the samples, the permutational multivariate analysis of variance (PERMANOVA), a non-parametric multivariate statistical permutation test, was performed; these analyses were performed using PRIMER v7 (PRIMER-E, Ltd). Inter-community and water mass differences in the abundance of each species were tested using one-way ANOVA and the Tukey–Kramer test; these analyses were performed using STAT-View.

## RESULTS

### Hydrography

**Fig. 2:**
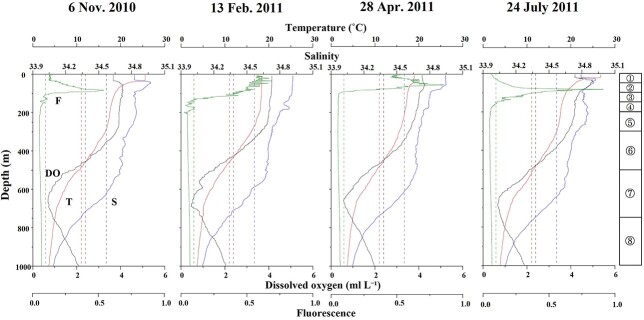
Vertical changes in temperature (T), salinity (S), DO (O₂), and fluorescence (F) at St. S1 during the sampling period from November 2010 to July 2011. The dotted guidelines show the mean value of all data for each parameter (T:11.00°C, F: 0.097, DO: 3.34 mL L^−1^, S: 34.4). Circled numbers in the right column indicate the depth strata of the zooplankton sampling in this study.

The vertical changes in temperature, salinity, DO and fluorescence for each sampling date at St. S1 are shown in Fig. 2. Throughout the study period, the temperature, salinity, DO and fluorescence ranged from 3.5–26.8°C, 34.0–34.9, 0.8–5.4 mL L^−1^ and 0.02–0.91 mg m^−3^, respectively. Regarding the temperature, the seasonal thermocline developed at ⁓50 m in November and July. In February and April, the temperature was almost uniform at 200–300 m, decreased gradually at 100–300 m and rapidly since 300–600 m and then smoothly decreased by 2–5°C at depths below 600 m. The salinity reached a maximum at ⁓50 m in November and July. Throughout the year, the salinity decreased gradually with depth between 100 and 300 m, reaching the minimum (ca. 34.0) level at ⁓600–700 m, and showed an increase with increasing depths beyond 700 m. The DO decreased with increasing depth. The rate of decrease in the DO was high at depths below 600 m. An oxygen minimum layer (OML) of < 2 mL L^−1^) was observed below 800 m. The fluorescence was high at depths of 0–200 m throughout the year. In February, the fluorescence was high at depths of 0–100 m. In other seasons, maximum subsurface fluorescence was observed near 100 m.

**Fig. 3:**
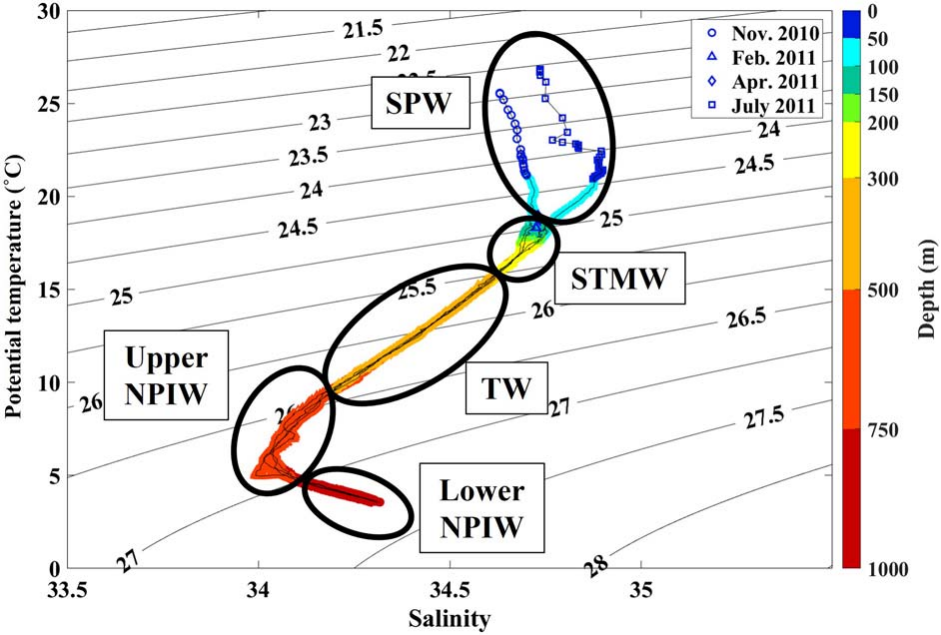
T–S diagram for the 0–1000 m water column at St. S1 during November 2010, February, April and July 2011. Five water masses were identified: SPW, STMW, TW, Upper NPIW and Lower NPIW. Solid lines on the background are isopycnals. Differences in the colors of the symbols denote differences in the zooplankton sampling layers.

The T–S diagrams for each sampling date are shown in Fig. 3. In all seasons, water masses characteristic of the western North Pacific subtropical region were observed. Four water masses were identified vertically: the shallowest SPW, STMW, NPIW including upper and lower NPIW, and TW present between the STMW and NPIW. The seasonal differences were obscured except for the seasonal thermocline at above ~ 100 m.

### Pelagic polychaete abundance and biomass

We found 27 pelagic polychaete species belonging to 13 genera and six families at St. S1 (Table II). The polychaete group with large eyes, previously categorized as tribe Alciopini (Phyllodocidae), was highly diverse (14 species). The most abundant species was the *Tomopteris* spp. without tails (annual mean: 21 ind. 1000 m^−3^), which accounted for 59% of the total abundance. For species identification of *Tomopteris* spp. (Tomopteridae), the following three characteristics were the most important: (i) the presence or absence of first tentacular cirri between the horn-like antenna and cirri-like appendages, (ii) the presence or absence and position of glands in the rami and (iii) the presence or absence of a tail on the pygidium. Because the rami condition was not good in this study and we could not observe glands in the rami (chromophile glands, hyaline glands and rosette glands), we described *Tomopteris* species as *Tomopteris* spp. (without tail), *Tomopteris* sp.1 (without tail) *and Tomopteris* spp. (with tail).

**Table II:** List of the pelagic polychaete species identified in the 0–1000 m water column at St. S1 in the western subtropical North Pacific from November 2010 to July 2011. Annual mean abundance and biomass (mean ± 1 SE: ind. 1000 m^−3^ or mg WW 1000 m^−3^ at 0–1000 m water column), species composition (%) and depth ranges (m) of occurrence are shown

Family		Abundance			Biomass		Occurrence depth (m)
	Species	(ind. 1000 m^−3^)	(%)		(mg WW 1000 m^−3^)	(%)	
Amphinomidae							
	Amphinomidae spp.	0.38 ± 0.06	1.08		1.33 ± 0.13	0.51	300–750
Lopadorrhynchidae							
	*Lopadorrhynchus brevis* Grube, 1855	1.48 ± 0	4.24		12.93 ± 0	4.94	50–750
	*Lopadorrhynchus uncinatus* Fauvel,1915	0.54 ± 0.28	1.54		1.76 ± 0.37	0.67	100–750
	*Pelagobia longicirrata* Greeff, 1879	0.85 ± 0.30	2.42		1.73 ± 0.87	0.66	200–1000
	Lopadorrhynchidae spp.	0.71 ± 0.04	2.02		2.66 ± 2.59	1.01	50–1000
Phyllodocidae							
	*Alciopa reynaudii* Audouin & H Milne Edwards, 1833	0.34 ± 0	0.98		16.56 ± 0	6.32	0–50
	*Naiades cantrainii* Delle Chiaje, 1830	0.42 ± 0.18	1.21		20.55 ± 18.26	7.85	0–100
	*Plotohelmis tenuis* (Apstein, 1900)	0.33 ± 0	0.95		0.80 ± 0	0.31	150–200
	*Rhynchonereella fulgens* Greeff, 1885	0.48 ± 0.23	1.37		13.22 ± 8.82	5.05	0–100
	*Rhynchonereella gracilis* Costa, 1864	0.24 ± 0.04	0.68		1.44 ± 1.03	0.55	50–200
	*Rhynchonereella m*ö*bii* (Apstein, 1893)	0.25 ± 0	0.71		1.40 ± 0	0.53	150–200
	*Torrea candida* (Delle Chiaje, 1841)	0.32 ± 0	0.91		4.55 ± 0	1.74	50–100
	*Vanadis formosa* Claparéde, 1870	0.74 ± 0.15	2.11		12.47 ± 4.11	4.76	0–150
	*Vanadis longissima* (Levinsen, 1885)	0.26 ± 0	0.74		18.32 ± 0	7.00	50–100
	*Vanadis* spp.	0.32 ± 0	0.91		14.05 ± 0	5.36	100–150
	Alciopini sp.1	0.31 ± 0	0.90		1.05 ± 0	0.40	100–150
	Alciopini sp.2	0.29 ± 0.01	0.90		0.826 ± 0.826	0.32	0–100
	Alciopini sp.3	0.58 ± 0.18	1.67		7.74 ± 5.68	2.96	0–200
	Alciopini spp.	0.56 ± 0.15	1.60		4.74 ± 2.67	1.81	0–150
Polynoidae							
	*Lepidasthenia grimaldii* (Marenzeller, 1892)	0.45 ± 0.11	1.29		3.67 ± 0.89	1.40	50–1000
Tomopteridae							
	*Tomopteris* spp. (without tail)	20.56 ± 2.56	58.7		46.10 ± 7.99	17.60	0–1000
	*Tomopteris* sp.1 (without tail)	0.33 ± 0	0.95		14.27 ± 0	5.45	100–200
	*Tomopteris* spp. (with tail)	0.67 ± 0.19	1.91		32.15 ± 11.64	12.27	0–1000
Typhloscolecidae							
	*Sagitella kowalewskii* Wagner, 1872	0.85 ± 0.26	2.43		9.38 ± 1.51	3.58	0–300
	*Travisiopsis levinseni* Southern, 1910	0.29 ± 0.01	0.84		3.77 ± 0.14	1.44	50–300
	*Travisiopsis lobifera* Levinsen, 1885	1.42 ± 0.61	4.05		9.05 ± 3.12	3.45	0–500
	*Typhloscolex muelleri* Busch, 1851	1.04 ± 0.36	2.98		5.41 ± 1.15	2.07	0–1000

**Fig. 4:**
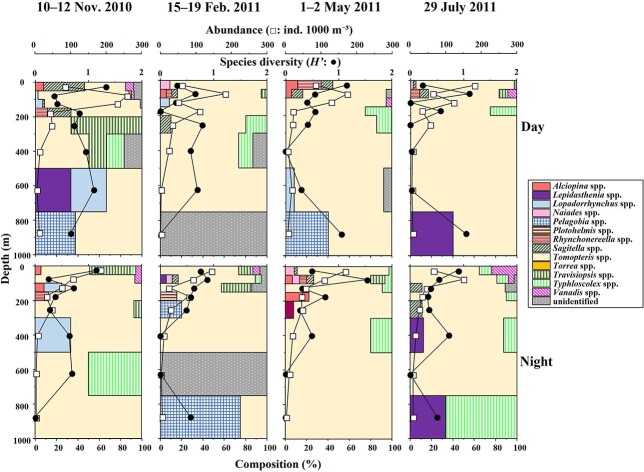
Vertical changes in polychaete abundance (ind. 1000 m^−3^), species composition, and species diversity (*H′*) in the 0–1000 m water column at St. S1 from November 2010 to July 2011. The composition of each genus is shown with a different color.

The abundance of pelagic polychaetes in the water column ranged from 1 to 260 ind. 1000 m^−3^ (Fig. 4). Throughout the days and nights of all seasons, the highest abundance was observed at depths of 0–200 m, with the maximum reported in November.

In terms of biomass, the *Tomopteris* spp. without a tail were also dominant, but to a limited extent (18% of the total pelagic polychaete biomass) (Table II). The biomass composition of the large *Tomopteris* spp. with a tail was high (12%), while their abundance was low (1.9%).

**Fig. 5:**
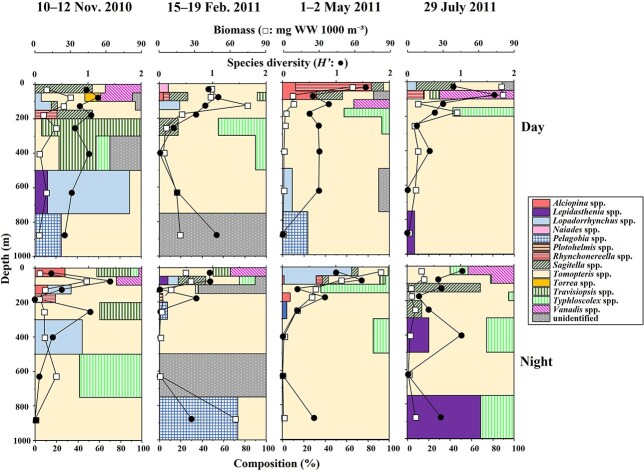
Vertical changes in polychaete biomass (wet weight: mg WW 1000 m^−3^), species composition and species diversity (*H′*) in the 0–1000 m water column at St. S1 from November 2010 to July 2011. The composition of each genus is shown with a different color.

The WW biomass ranged between 0.1 and 83.2 mg WW 1000 m^−3^ (Fig. 5). Increased biomass in the daytime was observed at 0–200 m for all three seasons, except for July. During the daytime in July, the highest biomass was observed at 750–1000 m because of the distribution of large *Tomopteris* spp. (with tail). At night, the highest biomass was observed at 0–200 m, especially in the shallowest layer of 0–50 m in November, February and May.

The species diversity (*H′*) of the pelagic polychaetes in the layers was 0–1.5 (based on abundance) and 0–1.6 (based on biomass), and the maximum *H′* was usually observed in the surface layer with no evident seasonal patterns (Figs 4 and 5). The *H′* was 0.47–1.01, based on the abundance in the samples.

A comparison of species abundance in each water mass showed that there was no significant difference in the abundances of species between different water masses, except for *Vanadis formosa* and *Sagitella kowalewskii* (Table III) that were abundant in the surface water mass, SPW and STMW (*P* < 0.05). Fifteen species (12 species belonging to Alciopini [tribe]) were distributed only at SPW or STMW, although not at a significant level.

**Table III:** Comparison of polychaete abundances for the five water masses (SPW, STMW, TW, Upper NPIW, Lower NPIW) at St. S1. Mean abundances for each water mass are shown. Differences between water masses were tested by one-way ANOVA and Tukey–Kramer test. For the results of the Tukey–Kramer test, differences in superscript letters indicate significant differences (*P* < 0.05). Numbers in the parentheses indicate the number of samples included in each water mass

Species	Abundance (ind. 1000 m^−3^)					one-way ANOVA
	SPW (8)	STMW (34)	TW (16)	Upper NPIW (10)	Lower NPIW (8)	
*Lopadorrhynchus brevis*	0.79	0.46	0.10	0.15	0	NS
*Lopadorrhynchus uncinatus*	0	0.49	0.22	0.13	0	NS
*Pelagobia longicirrata*	0	0.29	0.23	0	1.20	NS
*Alciopa reynaudii*	0.86	0	0	0	0	NS
*Naides cantraini*	0.56	0.87	0	0	0	NS
*Plotohelmis tenuis*	0.00	0.20	0	0	0	NS
*Rhynchonereella fulgens*	0.65	0.69	0	0	0	NS
*Rhynchonereella möebii*	0	0.15	0	0	0	NS
*Rynchonereella gracilis*	0	0.28	0	0	0	NS
*Torrea candida*	0.79	0	0	0	0	NS
*Vanadis formosa*	6.17^a^	0.72^b^	0^b^	0^b^	0^b^	***
*Vanadis longissima*	0.65	0	0	0	0	NS
*Alciopa* sp.1	0	0.18	0	0	0	NS
*Alciopa* sp.2	0.75	0.16	0	0	0	NS
*Alciopa* sp.3	0.75	0.85	0	0	0	NS
*Lepidasthenia grimaldii*	0	0.21	0.22	0.46	0.39	NS
*Tomopteris* sp.1	0	0.31	0	0	0	NS
*Sagitella kowalewskii*	4.95	2.14	0	0	0	*
*Travisiopsis levinseni*	0	0.26	0	0	0	NS
*Travisiopsis lobifera*	6.63	2.10	0.14	0	0	NS
*Typhloscolex muelleri*	3.87	1.44	0.91	0.75	0.77	NS

^*^ *P* < 0.05; ^***^P < 0.001; NS: not significant; Alphabets on values show group.

### Pelagic polychaete community structure

**Fig. 6:**
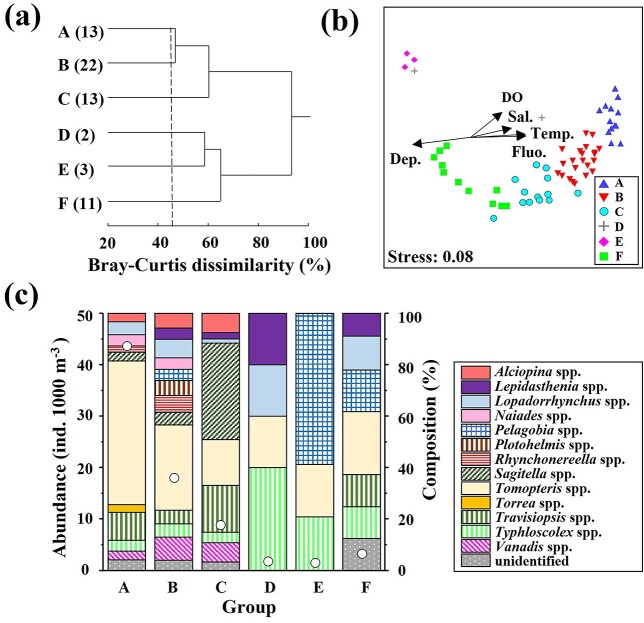
**(a)** Results of cluster analysis based on pelagic polychaete abundance at St. S1. Six groups (A–F) were identified at 45% Bray–Curtis dissimilarity connected with the complete linkage method. Numbers in parentheses indicate the number of samples each group contained. **(b)** NMDS plots of each group. Arrows indicate directions of significant environmental parameters. Dep.: depth, Temp.: temperature, Sal.: salinity, DO: dissolved oxygen, Fluo.: fluorescence. Stress is a value used to evaluate the quality of NMDS, which is low for relevant NMDS. **(c)** Mean abundance (white circle) and genus composition of each group.

Based on abundance, the pelagic polychaete community was clustered into six community groups (A–F) with a 45% dissimilarity (Fig. 6a). Each group contained 2–22 samples. NMDS separated the groups, and various environmental factors (depth, salinity, DO and fluorescence) were found to have significant directions according to Pearson’s multidimensional coefficient. From the plotted directions, depth and DO were related to pelagic polychaete clustering. Depth and environmental factors depending on the depth (salinity, temperature and fluorescence) affect horizontally, and the clustering groups are plotted separately in the horizontal direction. DO had a significant effect on the grouping of groups A (high DO), B (moderate DO) and C (low DO) (Fig. 6b). The mean abundance of group A (43.9 ind. 1000 m^−3^) was higher than that of groups B (18.3 ind. 1000 m^−3^) and C (9.1 ind. 1000 m^−3^), with *Tomopteris* spp. (without tail) accounting for 56%. *Tomopteris* spp. (without tail) was dominant in group B, while *Sagitella* spp. was dominant in group C (Fig. 6c). Low abundances were observed in groups D, E and F. Different species compositions were observed in these groups. *Typhloscolex muelleri* was dominant in group D, *Pelagobia longicirrata*, in group E and *Travisiopsis* spp., only in group F. The numbers of genera differed within the groups (3–11 genera), and some genera occurred in specific groups (*Alciopina* spp.: A–C, *Plotohelmis* spp.: B, *Rhynchonereella* spp.: A and B, *Sagitella* spp.: A–C, *and Torrea* spp.: A) (Fig. 6c). Most of them (except *Sagitella* spp.) belonged to the Alciopini (tribe). Inter-group differences in abundance were detected only for *V*. *formosa,* which was abundant in group B (*P <* 0.05, Table IV).

**Table IV:** Comparison of the polychaete abundances in the six groups (A–F) identified by Bray–Curtis dissimilarity (cf. Fig. 6a). Mean abundances for each group are shown. Differences between groups were tested by one-way ANOVA and Tukey–Kramer test. For the results of the Tukey–Kramer test, differences in superscript letters indicate significant differences (P < 0.05). Numbers in the parentheses indicate number of samples included in each group

Species	Abundance (ind. 1 000 m^−3^)						one-way ANOVA
	A (13)	B (22)	C (13)	D (2)	E (3)	F (11)	
*Lopadorrhynchus brevis*	11.02	0	0	0	0	0	NS
*Lopadorrhynchus uncinatus*	0	8.36	1.29	0	0	2.29	NS
*Pelagobia longicirrata*	0	4.95	0	0	3.90	2.84	NS
*Alciopa reynaudii*	6.84	0	0	0	0	0	NS
*Naides cantraini*	9.34	5.09	0	0	0	0	NS
*Plotohelmis tenuis*	0	6.69	0	0	0	0	NS
*Rhynchonereella fulgens*	0	9.57	0	0	0	0	NS
*Rhynchonereella möebii*	0	4.94	0	0	0	0	NS
*Rynchonereella gracilis*	5.60	3.98	0	0	0	0	NS
*Torrea candida*	6.34	0	0	0	0	0	NS
*Vanadis formosa*	7.50^b^	15.43^a^	5.99^b,c^	0^b,c^	0^b,c^	0^c^	*
*Vanadis longissima*	0	5.18	0	0	0	0	NS
*Alciopa* sp.1	0	6.29	0	0	0	0	NS
*Alciopa* sp.2	5.60	0	5.99	0	0	0	NS
*Alciopa* sp.3	9.23	6.59	5.99	0	0	0	NS
*Lepidasthenia grimaldii*	0	5.06	1.97	1.53	0	1.56	NS
*Tomopteris* sp.1	3.92	6.69	0	0	0	0	NS
*Sagitella kowalewskii*	7.43	5.66	29.96	0	0	0	NS
*Travisiopsis levinseni*	5.60	0	3.09	0	0	0	NS
*Travisiopsis lobifera*	33.06	6.01	20.12	0	0	2.18	NS
*Typhloscolex muelleri*	9.33	5.93	3.30	3.07	1.39	2.18	NS

^***^ *P* < 0.001; ^*^P < 0.05; NS: not significant; Alphabets show group.

**Fig. 7:**
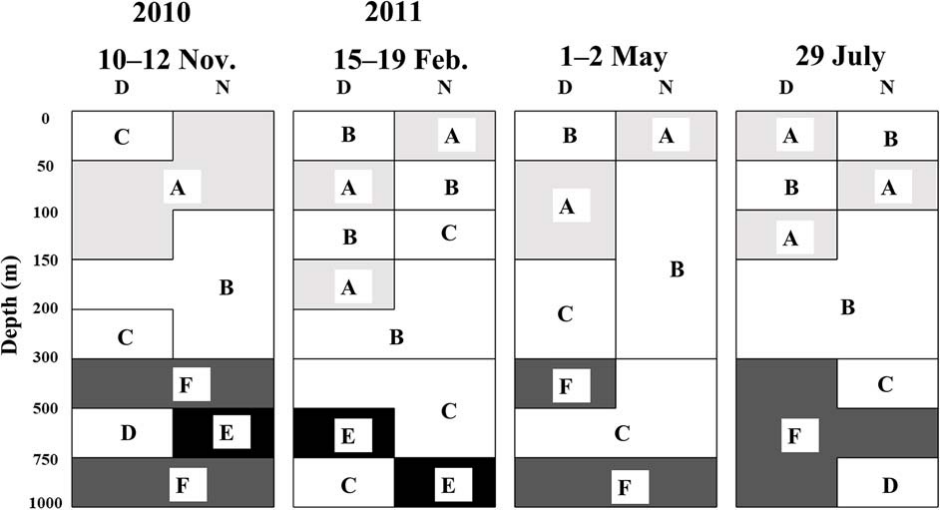
Seasonal, vertical, and diel changes in the occurrence of the five polychaete community groups (A–F) identified by Bray–Curtis dissimilarity based on their abundance at St. S1 from November 2010 to July 2011 (cf. Fig. 6a). D: day, N: night.

The diel, vertical and seasonal distributions of pelagic polychaete community groups at St. S1 are shown in Fig. 7. The occurrence of each group was governed by depth. The high-abundance groups (A and B) were observed at depths of 0–300 m. Group C was mainly observed at depths of 200–750 m. The occurrence of low-abundance groups (D, E and F) was restricted to depths below 300 m. Thus, the distribution of the pelagic polychaete community primarily varied vertically and did not show diel or seasonal patterns. The results of the PERMANOVA also indicated that depth was the most prominent environmental factor in polychaete grouping (*P <* 0.001) (Table V). The interaction between season×day/night also had a significant effect (*P <* 0.05).

**Table V:** PERMANOVA results based on Bray–Curtis dissimilarities using the polychaete abundance data in relation to the environmental factors at St. S1 (season, day/night and depth). D: day, N: night

Factors	*df*	Pseudo *F*	*P*
Season	3	1.3757	ns
D/N	1	0.72803	ns
Depth	7	4.277	<0.001
Season×D/N	3	1.534	<0.05
Season×Depth	21	1.1058	ns
D/N × Depth	7	0.98467	ns

## DISCUSSION

### Fauna and species diversity

In the western North Pacific, the number of pelagic polychaete species at subtropical St. S1 (27 species) (Table II) was much higher than that at subarctic St. K2 (10 species) (Amei *et al.*, 2021). Four common families were present in both regions: Lopadorrhynchidae, Phyllodocidae, Typhloscolecidae and Tomopteridae. Iospilidae and Flabelligeridae occurred only in the subarctic region, whereas Amphinomidae and Polynoidae were present only in the subtropical region. Fernández-Álamo *et al.* (2003) reported a clear latitudinal pattern reported for the 0–200 m water column between 21°N and 40°N latitudes off California, eastern North Pacific, based on CalCOFI samples. According to Fernández-Álamo *et al.* (2003), the pelagic polychaete community can be divided into the north, south and transition groups. The north group is characterized by high abundance and relative diversity (11 species), the south group is characterized by low abundance but the most diverse (16 species), and the transition group has the lowest abundance and species diversity (nine species). St. S1 is in 30°00’N, which is included in the transition group of Fernández-Álamo *et al.* (2003). However, the transition group showed a similar pelagic polychaete community with the north group and different with St. S1. It suggests that the pelagic polychaete distribution pattern strongly depends on water flow rather than latitude. Cold California Current flows from north to south along California, and warm Surface Equatorial Water (temperature > 20°C, salinity < 35 in their study) existed only in the southern region (⁓21–25°N corresponding to St.32–35) (Fig. 2, Fernández-Álamo *et al.*, 2003). Among the three groups, a south group characterized by the presence of subtropical pelagic polychaete genera such as *Vanadis*, *Rhynchonerella* and *Plotohelmis* had the most similar community with that of St. S1 reported in this study. The high diversity shown in St. S1 suggests the presence of a difference in pelagic polychaete community structure between the west and east region of the subtropical gyre in the Pacific Ocean.

**Fig. 8:**
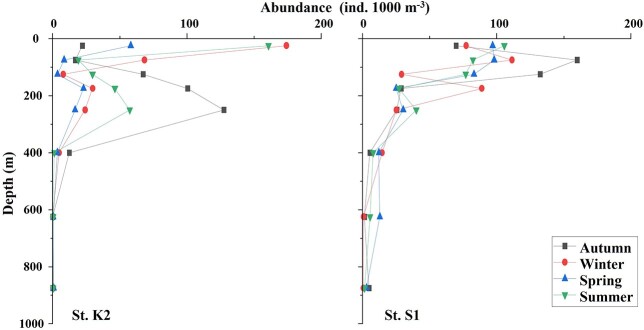
Seasonal changes in the vertical distribution for the mean abundance of *Tomopteris* species in the subarctic and subtropical regions (K2: *T. septentrionalis*, S1: *Tomopteris* spp.).

Tebble (1962) reported seven tomopterid species from a wide region of the Pacific Ocean, including the sampling region of this study. He also mentioned the important morphologic traits for Pacific tomopterid identification. According to him, [*Tomopteris* spp. with tail] includes *Tomopteris nisseni*, *Tomopteris krampi*, *Tomopteris apsteini, Tomopteris pacifica*, and [*Tomopteris* spp. without tail] includes *Tomopteris planktonis*, *Tomopteris elegans*, *Tomopteris ligulata* and *Tomopteris septentrionalis*. In subtropical S1, *Tomopteris* spp. (with and without tail), with high proportions throughout the water column, were concentrated at the surface layer. Tomopteridae, a common family worldwide, is reported to dominate the Pacific Ocean, especially in subtropical and subarctic regions (e.g. Tebble, 1962). In the present study, subtropical *Tomopteris* spp. (without tail) were too small to preserve the rami. In both regions, this family was distributed widely at 0–1000 m, and peaks in its abundance were observed at depths shallower than 300 m. *Tomopteris* spp. (without tail) at S1 showed no seasonal changes in vertical distribution (Fig. 8), in contrast to *T. septentrionalis* at K2. Unfortunately, in this study, we cannot treat *Tomopteris* spp. (without tail) as one species. However, the difference in the vertical distribution between these subtropical and subarctic dominant *Tomopteris* species suggests that *Tomopteris* species have different dynamics depending on marine environmental factors, such as the concentration of chlorophyll *a* in each region.

Within the four families commonly occurring in both regions, the Alciopini (tribe), belonging to Phyllodocidae, showed substantial diversity in the subtropical region. Thus, while only two species were observed in the subarctic region, 14 were in the subtropical region. Phyllodocidae, including Alciopini, was the second most dominant family in the subtropical region (Table II). Alciopid polychaetes are abundant and adapted to tropical/subtropical regions with eyes equipped with morphologically specialized lenses (Dales, 1957; Ushakov, 1974). The eyes of alciopid polychaetes have accessory retinas beside the lens that can even detect ultraviolet light (Wald and Rayport, 1977). These facts suggest that alciopids are well adapted to high light conditions under low light attenuation in the oligotrophic tropical/subtropical region. Thus, active visual-predator alciopids may have more opportunities to prey under sufficient light with high light radiation and transmission in the subtropical region than in the subarctic region. These characteristics of alciopids (high swimming ability and specialized eyes) may allow for their high diversity and numerical dominance in the pelagic polychaete community in the subtropical region.

*Pelagobia longicirrata* was a common species in both regions. *Pelagobia longicirrata* has been treated as a cosmopolitan species; however, Kolbasova and Neretina (2021) reported that *P. longicirrata* has some geographically different clades in a phylogenetic tree based 28S and 18S. There is no phylogenetic information on *P. longicirrata* in the Pacific Ocean. In the subtropical region, *P. longicirrata* accounted for only 2% of the abundance and was vertically concentrated at depths greater than 750 m (Table II). This species is the most abundant in the subarctic region, accounting for 74% of the pelagic polychaete abundance (Amei *et al.*, 2021). In both regions, *P. longicirrata* was distributed at depths greater than 150 m. They showed distribution at a wide depth range (150–1 000 m) at subarctic St. K2 and were concentrated to the deepest layer (750–1000 m) at subtropical St. S1. Thus, the distribution of *P. longiciratta* ranged from the subarctic region (below 150 m) to the subtropical region (in the NPIW layer). The abundance and biomass of *P. longicirrata* in the subtropical region were much lower than those in the subarctic region. It may be related to the lower primary production in the subtropical region than in the subarctic region.

**Fig. 9:**
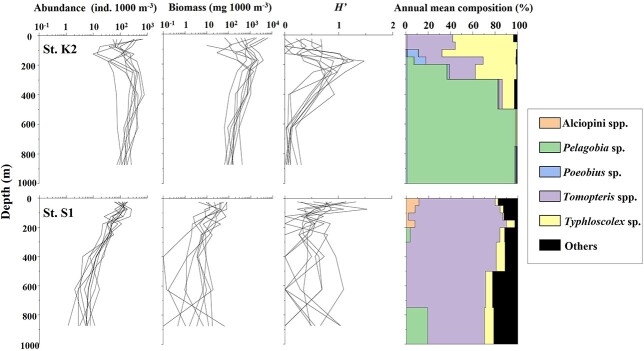
Vertical changes in the polychaete abundance, biomass, species diversity (*H′*) and genus composition in annual mean abundance (%) for the 0–1000 m water column in the subarctic (St. K2) and the subtropical (St. S1) regions in western North Pacific. Lines represent values for days and nights during the four seasons from 2010 to 2011.

For various zooplankton taxa, higher species diversity has been reported in the subtropical regions than in the subarctic areas (e.g. Tittensor *et al.*, 2010; Sun and Wang, 2017). Sun and Wang (2017) showed the change of diversity *H′* on the transect of 160°E from 4°S to 46°N based on zooplanktons sampled by 200 μm-mesh two-paralleled WP2 net from 200 m depth. They reported a rapid decrease of *H′* between 30°N and 40°N. However, the *H′* values of the pelagic polychaetes throughout each layer were similar (0.82–1.17) at K2 and (0.47–1.01) at S1. While the *H′* values were similar for both regions, it should be noted that the vertical distribution of *H′* varied with the region. Thus, *H′* peaked at 100–300 m at St. K2, while peaks formed at the surface layer at St. S1 (Fig. 9). These results reflect regional differences in pelagic polychaete communities. At St. K2, *T. septentrionalis* and *T. muelleri* dominated at 0–100 m, *P. longicirrata* dominated at the deeper layers below 300 m, and the *H′* peaked at 100–300 m depth where all the species occurred (Fig. 9). At St. S1, the vertical distribution of pelagic polychaetes was concentrated in the surface layer. The *H*′ values also peaked near the surface layer (Fig. 9). At both stations, vertical patterns of *H′* were similar to that of pelagic polychaete abundance. It suggests that the diversity of rare taxa pelagic polychaetes strongly depends on their abundance.

### Abundance and biomass

**Fig. 10:**
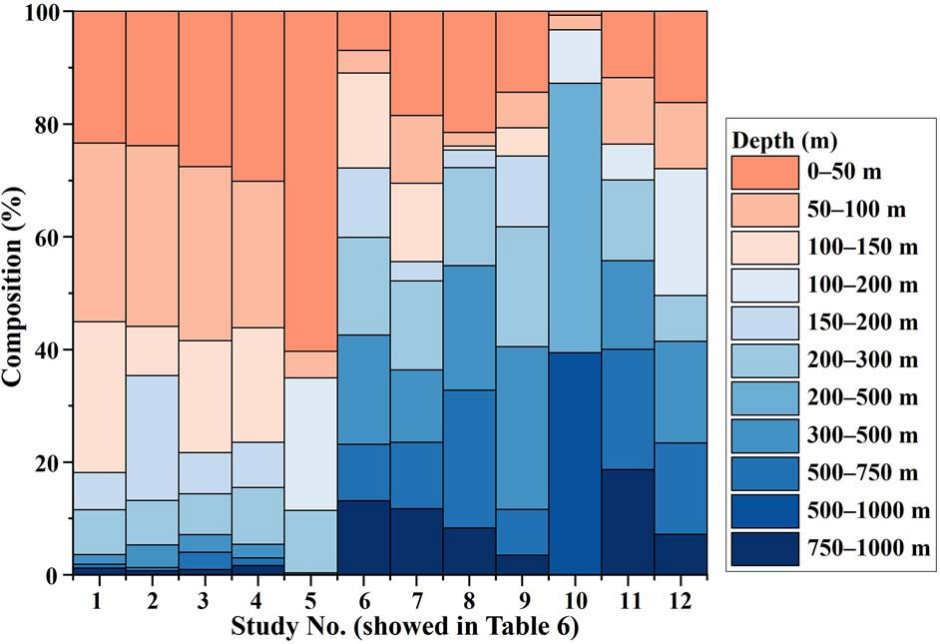
Percentage composition of sampling layers in pelagic polychaete distribution within 0–1 000 m water column reported by this study and previous studies. Study No. corresponds to Table VI. No. 1–5 research in the subtropical region and the rest in the subarctic region of the Pacific Ocean.

Annual mean abundance and biomass of the pelagic polychaetes in the subtropical region were 35.0 ind. 1000 m^−3^ and 17.3 mg WW 1000 m^−3^, respectively (Table II). These values are much lower than those in the subarctic region (239.2 ind. 1000 m^−3^ and 517.5 mg WW 1000 m^−3^; Amei *et al.*, 2021) (Fig. 9). The differences between the regions (S1:K2) were expressed as a factor of 0.146 (abundance) or 0.033 (biomass). The vertical distributions of abundance also varied with the region. The vertical patterns of pelagic polychaete abundance at St. K2 resembled a gradual arch-shape with peaks from 200 to 500 m depth (Amei *et al.*, 2021). On the other hand, that at St. S1 decreased increasing depth. For comparison of the vertical pattern of pelagic polychaete community structures between the subtropical and subarctic Pacific Ocean, we listed a diel-mean abundance of pelagic polychaetes for each depth layer shown by this study and comparable previous studies (Table VI). Steinberg *et al.* (2008) reported vertical patterns of some zooplankton taxa at subtropical St. ALOHA and subarctic St. K2, and Mileykovskiy (1969) reported the on pelagic polychaetes at Kuril Kamchatka Trench. Excluding Mileykovskiy (1969), all studies used IONESS or MOCKNESS with 335 μm for sampling. The percentages of distributing depth of pelagic polychaetes are similar between this study and the previous studies (Fig. 10).

**Table VI:** Diel-mean abundances of pelagic polychaetes for 0–1000 m depth reported by this study and comparable previous studies conducting vertically stratified sampling into ≥1000 m depth in the subtropical and subarctic Pacific Ocean

Location	Subtropic region in the western Pacific Ocean (St. S1)				Subarctic region in the western Pacific Ocean (St. K2)				Kuril Kamchatka Trench		St. K2	St. ALOHA
Study No.	1	2	3	4	6	7	8	9	10	11	12	5
Season	Nov.	Feb.	May	July	Oct.	Feb.	April	July	1. Aug.	29–30. Aug.	22. July –11. Aug.	22. Jun. –9. July
Sampling layers	Abundance (ind. 1000 m^−3^)											
0–50 m	134	103	128	124	114	202	375	345	36	380	450	950
50–100 m	182	138	143	107	66	131	43	151	139 Incomplete	380	325	75
100–150 m	153	37	92	84	275	151	12	121	513	206	625	370
150–200 m	38	96	34	33	202	38	54	301				
200–300 m	46	34	34	41	284	172	305	512	2 580	460	225	175
300–500 m	10	17	14	10	317	140	386	694		509.5	500	5
500–750 m	4	2	14	6	164	129	428	195	2 129	688	450	0.5
750–1 000 m	7	3	5	7	216	128	145	85		603	200	0.25
Net	IONESS net (335 μm)				IONESS net (335 μm)				Juday (DZhOM 80/113) net of No. 38 mesh		IONESS (335 μm)	MOCNESS (335 μm)
Reference	This study				Amei et al., 2021				Mileykovskiy, 1969		Steinberg et al., 2008	

Kitamura *et al.* (2016), reporting biomass data of mesozooplankton in the same samples as this study, suggested the occurrence of diel vertical migration (DVM) and seasonality at St. S1 related to the primary production. However, pelagic polychaetes at St. S1 did not show significant DVM and seasonality. A pelagic polychaete DVM cannot be detected because of the surface layer limited distribution of pelagic polychaetes in the subtropical Pacific Ocean, especially Alciopini. It is also possible that pelagic polychaete DVM was too small to detect by the resolution of sampling layers in this study. Besides, the seasonality of this study’s pelagic polychaetes of St. S1 can be related to their reproduction and ecology. However, almost all of them remain unknown, especially for subtropical species.

Given the regional differences in mesozooplankton stocks in the western North Pacific, the annual mean biomass of the whole zooplankton population at St. K2 (5.8 mg C m^−2^) has been reported to be ten times higher than that at St. S1 (0.52 mg C m^−2^) (Kitamura *et al.*, 2016). Further, the zooplankton biomass at 0–1 000 m depths was greater at St. K2 than at the subtropical St. ALOHA station (Steinberg *et al.*, 2008). Based on the contents of the sediment trap moored at 200 m depth at St. K2 and S1, zooplankton fluxes in number and mass have also been reported to be 2.3–6.5 times higher at St. K2 than at St. S1 (Yokoi *et al.*, 2018). The flux of pelagic polychaetes was also analyzed for these sediment trap samples, and their annual mean values were 5.37 ind. m^−2^ day^−1^ at St. K2 and 1.36 ind. m^−2^ day^−1^ at St. S1 (Amei *et al.*, 2020). Thus, the abundance and biomass were several times greater in the subarctic region than in the subtropical area, which are common phenomena for pelagic polychaetes and whole zooplankton.

In the K2S1 project, which focused on regional comparisons of carbon cycles, the standing stocks (mg C m^−2^) and fluxes (mg C m^−2^ day^−1^) of various biogeochemical components have been quantified (Honda *et al.*, 2016, 2017). While the standing stocks and fluxes of most components were at similar levels in both regions, the standing stocks of metazooplankton were reported to be ten times greater in the subarctic region than in the subtropical region (Honda *et al.*, 2016, 2017; Matsumoto *et al.*, 2016; Wakita *et al.*, 2016). To explain the high mesozooplankton standing stocks, Kitamura *et al.* (2016) proposed three factors: (i) The respiratory and excretory carbon losses per unit biomass due to large organisms were lower than those due to small organisms (mesozooplankton at the K2 were larger than those at the S1), (ii) the lower temperature at St. K2 allowed the mesozooplankton to decrease respiratory carbon loss and (iii) the higher turnover rates of small organisms at St. S1 may have helped maintain their biomass to low levels. The energy loss from primary producers to the mesozooplankton community at St. K2, in which primary consumers were probably dominant, was considered to be lower than the community at St. S1. The regional patterns of the standing stocks of pelagic polychaetes fit well with the mesozooplankton stocks in each region. The difference in the body lengths of dominant *Tomopteris* species (without tail) was detected between the subarctic and subtropical regions, and this may have led to variations in the standing stocks between St. S1 and K2 (Kitamura *et al.*, 2016). To mention pelagic polychaetes for other discussions (2) and (3) in Kitamura *et al.* (2016), we need more information on their life cycle, metabolic rate and food.

### Factors affecting community structure

According to the results of multiple regression analysis and PERMANOVA, the most important factor affecting the clustering of the pelagic polychaete community in the subtropical region was depth (*P <* 0.001, Table V). In the subarctic region, the season was also found to affect the pelagic polychaete community in addition to season (*P <* 0.05; Amei *et al.*, 2021). This may be related to the seasonal occurrence of the community in which *P. meseres* was the dominant species, which feeds on sinking particles (detritus) and is abundant in the 100–300 m depths during spring and summer when a large amount of sinking particle detritus is available (Amei *et al.*, 2021). For both regions, interactions related to day or night (×D/N) had a significant effect on the polychaete community (Table V), possibly owing to the effect of DVM of surface-dwelling carnivorous species (e.g. *Tomopteris* spp.) in each region.

The abundance and biomass of pelagic polychaetes in subtropical S1 were concentrated at 200–300 m occupied with STMW and decreased rapidly at deeper layer occupied with TW and NPIW (Figs. 3 and 4). SPW was only observed in November and July. However, no differences in pelagic polychaete abundance and composition on the surface layer between the seasonal thermocline season (November, July) and non-seasonal thermocline season (February, April) were detected. This suggests that the seasonal thermocline cannot be a barrier to pelagic polychaete distribution at St. S1. The limited distribution of visual carnivore feeders, Alciopini (tribe) (cluster groups A–C) in surface water (SPW and STMW), is related to the vertical changes in the light transmission rate. Among the three groups, the group with the highest DO level had the highest mean abundance (group A > B > C) (Fig. 6c). Carnivores such as *Tomopteris* and Alciopini (tribe) dominate the surface group and have high metabolic rates for DVM, positive swimming and seeks prey (Childress and Thuesen, 1993; Thuesen and Childress, 1993). Besides, Joost *et al.* (2021) suggested that Tomopteridae need to stroke their parapodium when they swim actively. Thus, they can need high concentrations of DO to feed on their prey actively.

*Poeobius meseres* has unique feeding modes wherein it secretes mucus nets to capture the sinking food particles (detritus) (Robison *et al.*, 2010; Uttal and Buck, 1996); this species also has a low metabolic rate and engages in little swimming activity, which may be considered adaptations to the low oxygen concentration in the deep layer, i.e. the OML (Childress and Thuesen, 1993; Thuesen and Childress, 1993). In the subarctic and subtropical regions, OML, which is a prominent environmental parameter for vertical changes, is observed below 200 m (Fig. 2). From the viewpoint of adaptation to the OML, the low metabolic rates of *P. meseres* (Childress and Thuesen, 1993; Thuesen and Childress, 1993) may facilitate its distribution in the OML. Notably, *P. meseres* occurs only in the deep layer of the subarctic region (Amei *et al.*, 2021). Because the vertical flux of fecal pellets is reported to be much higher in the subarctic region than in the subtropical region (Kobari *et al.*, 2016), the increased food availability may also support the distribution of *P. meseres* in the subarctic region. In the pelagic polychaete community, carnivorous pelagic polychaetes dominated the surface of both the subarctic and subtropical regions. However, the compositions of deep-sea communities varied regionally. The scarcity of sinking particles and the low availability of fecal pellet flux in the subtropical region (Kobari *et al.*, 2016) may prevent the distribution of particle feeders in the deep layer of the subtropical region. In the deep layer of the subarctic region, a unique, specialized community of pelagic polychaetes adapted to low oxygen conditions is supported by the abundant nutrient flux via sinking particles and zooplankton fecal pellets. Thus, the two regions differed in the pelagic polychaete community structures and the vertical patterns.

## CONCLUSIONS

This study was conducted for latitudinal comparison with the results of Amei *et al.* (2021), which revealed diel, seasonal, and vertical changes in the pelagic polychaete community at St. K2 in the western subarctic Pacific. By applying the same sampling and analytical methods as Amei *et al.* (2021) at St. S1 in the western subtropical Pacific, we evaluated several latitudinal differences in the pelagic polychaete community. First, the numbers of genera and species were higher at St. S1 than at St. K2. The annual mean abundance and biomass were much higher at St. K2. These regional patterns were attributed to regional differences in productivity, temperature-induced metabolism and community structure at each region. At St. S1, carnivorous pelagic polychaetes were dominant throughout the sampling layers and concentrated in the surface layer, and their abundance rapidly decreased with increasing depth. On the other hand, at St. K2, numerous particle-feeding species were present in the pelagic polychaete community, and their abundance peaked at a depth of 300 m, with the predominance of particle feeders at the deep layer of the station; this finding can be attributed to the passive flux of sinking particulate matter, such as fecal pellets, at St. K2. The pelagic polychaete community structures varied depending on depth, and they differed between the two regions.

## Data Availability

Data will be provided by the corresponding author on request.
